# Variations and obstacles in the use of coagulation factor concentrates for major trauma bleeding across Europe: outcomes from a European expert meeting

**DOI:** 10.1007/s00068-020-01563-2

**Published:** 2021-01-05

**Authors:** Vladimir Černý, Marc Maegele, Vanessa Agostini, Dietmar Fries, Santiago R. Leal-Noval, Gábor Nardai, Giuseppe Nardi, Anders Östlund, Herbert Schöchl

**Affiliations:** 1 Department of Anesthesiology, Perioperative Medicine and Intensive Care, JE Purkinje University, Usti Nad Labem, Masaryk Hospital, Prague, Czech Republic; 2 Department of Trauma and Orthopedic Surgery, Institute for Research in Operative Medicine (IFOM), University Witten/Herdecke, Cologne-Merheim Medical Center (CMMC), Cologne, Germany; 3 IRCCS Ospedale Policlinico San Martino, Genova, Italy; 4 Department for General and Surgical Critical Care Medicine, Medical University Innsbruck, Innsbruck, Austria; 5 Critical Care Division, University Hospital ‘Virgen del Rocio’, Seville, Spain; 6 Péterfy Sándor Str. Hospital and Trauma Center, Budapest, Hungary; 7 Department of Anesthesia and Intensive Care, Rimini Hospital, Rimini, Italy; 8 Perioperative Medicine and Intensive Care, Karolinska University Hospital Solna, Stockholm, Sweden; 9 AUVA Trauma Centre Salzburg, Salzburg, Austria

**Keywords:** Trauma-induced coagulopathy, Bleeding, Coagulation factor concentrates, Fibrinogen concentrate, Expert opinion

## Abstract

**Purpose:**

Trauma is a leading cause of mortality, with major bleeding and trauma-induced coagulopathy (TIC) contributing to negative patient outcomes. Treatments for TIC include tranexamic acid (TXA), fresh frozen plasma (FFP), and coagulation factor concentrates (CFCs, e.g. prothrombin complex concentrates [PCCs] and fibrinogen concentrate [FCH]). Guidelines for TIC management vary across Europe and a clear definition of TIC is still lacking.

**Methods:**

An advisory board involving European trauma experts was held on 02 February 2019, to discuss clinical experience in the management of trauma-related bleeding and recommendations from European guidelines, focusing on CFC use (mainly FCH). This review summarises the discussions, including TIC definitions, gaps in the guidelines that affect their implementation, and barriers to use of CFCs, with suggested solutions.

**Results:**

A definition of TIC, which incorporates clinical (e.g. severe bleeding) and laboratory parameters (e.g. low fibrinogen) is suggested. TIC should be treated immediately with TXA and FCH/red blood cells; subsequently, if fibrinogen ≤ 1.5 g/L (or equivalent by viscoelastic testing), treatment with FCH, then PCC (if bleeding continues) is suggested. Fibrinogen concentrate, and not FFP, should be administered as first-line therapy for TIC. Several initiatives may improve TIC management, with improved medical education of major importance; generation of new and stronger data, simplified clinical practice guidance, and improved access to viscoelastic testing are also critical factors.

**Conclusions:**

Management of TIC is challenging. A standard definition of TIC, together with initiatives to facilitate effective CFC administration, may contribute to improved patient care and outcomes.

## Introduction

Trauma remains one of the leading causes of morbidity and mortality, and injuries are responsible for ~ 5.8 million deaths annually, accounting for ~ 10% of deaths worldwide [1, 2]. Massive bleeding in traumatic injury represents a substantial problem, and is a major cause of potentially preventable deaths [3–5].

Trauma-induced coagulopathy (TIC) is present in approximately 24–34% of hospitalised patients with trauma [6, 7]. It comprises an endogenous impairment of haemostasis that occurs early after injury [7]. Failure to form haemostatic clots leads to coagulopathic bleeding, with diffuse phenotypes involving uninjured sites, and is difficult to stop with mechanical interventions [8]. The causes of TIC are multifactorial, with key drivers including shock, acidosis, endotheliopathy, and consumption/loss of coagulation factors (e.g. due to haemodilution) [8–12]. TIC is associated with increased mortality, transfusion requirements and multiple-organ failure [6, 9, 13]. While progress has been made on understanding the causes of TIC, it continues to present a significant clinical challenge, and a clinically relevant, uniformly accepted definition for TIC is lacking [8, 9].

This review focuses on treatments for TIC, which require different treatment strategies to those for other bleeding situations such as during surgery, that may require mechanical interventions [14]. Current TIC treatment options include tranexamic acid (TXA), fresh frozen plasma (FFP), cryoprecipitate and coagulation factor concentrates (CFCs) [10]. CFCs, including prothrombin complex concentrates (PCCs) and human fibrinogen concentrate (FCH), have several benefits over FFP in that they deliver a standardised and higher concentration of coagulation proteins, and are associated with a low risk of virus transmission and transfusion-related side effects, such as acute respiratory distress syndrome, sepsis and multiple organ failure. Furthermore, they are immediately available without requiring blood group testing [15, 16]. CFCs can be used for goal-directed therapy, an individualised point-of-care (POC) approach, using viscoelastic tests to elucidate potential haemostatic deficiencies [17]. The use of CFCs for the management of TIC has recently shifted into research focus; for example, several studies have documented a benefit with FCH, including lower mortality and/or reduced transfusion requirements, versus FFP/no fibrinogen supplementation [18–20]. However, a meta-analysis of seven RCTs found no beneficial effect on in-hospital mortality with FCH versus controls. Although data on FCH use in trauma are limited and of poor quality; most studies were retrospective, with varied endpoints [21]. Therefore, the beneficial effects of FCH on mortality still need to be elucidated.

Clinical strategies for TIC management, including the use of CFCs, are heterogeneous [22]. In Europe, four-factor PCCs are indicated for the reversal of vitamin K antagonists (VKAs), and in acquired deficiency of prothrombin complex coagulation factors, e.g. in trauma [23]. FCH is indicated for the treatment of acquired hypofibrinogenaemia across Europe, though in many countries, the indication is restricted to acquired hypofibrinogenaemia during surgical intervention [24, 25]. Guidelines for the management of bleeding have been published both Europe-wide [10] and locally [26–31]; however, national guideline recommendations for haemostatic management (for example, the use of CFCs and FFP) differ between countries, as do the availability and licensing of the products; therefore, there is a need to streamline clinical pathways to facilitate consistent and effective management.

The aims of this review are 1) to summarise recommendations from the available European trauma guidelines, with a focus on the use of CFCs (mainly FCH), highlighting the differences between the European versus local guidelines; 2) to provide a simple definition of TIC and the criteria for initiation of a massive trauma protocol (MTP), that can be easily interpreted in clinical practice; and 3) to identify gaps in the guidelines that impact on their application in daily clinical practice, as well as the barriers to effective CFC administration, while providing practical guidance and recommendations on how these challenges may be overcome.

## Methods

An advisory board was held on 2 February 2019, titled ‘The role of coagulation factor concentrates in the management of major trauma bleeding across Europe: an EU advisory board.’ The advisors, all experts in the fields of trauma and critical care medicine, discussed their clinical experience with CFCs (mainly FCH) in the management of trauma-related bleeding. The clinical application of the current European and local guidelines on trauma management was discussed. Evidence to support the use of CFCs in TIC, along with barriers to CFC use were also highlighted, with suggestions on how these barriers may be overcome.

A comprehensive literature search was conducted for articles on, or prior to, 19 April 2018, to identify guidelines and recommendations for fibrinogen supplementation and POC testing in the trauma setting, to support expert discussions. Google and PubMed searches were conducted using the search terms: ‘trauma bleeding guidelines,’ ‘trauma guidelines coagulation’ and ‘trauma management guidelines;’ country-specific terms were also added to identify local publications. The searches focused on the latest European guidelines published in English, supplemented with local European guidelines (as suggested by the advisors).

## Overview of published European guidelines for major bleeding and coagulopathy following trauma

The literature search identified the fifth edition of the European guidelines for major bleeding and coagulopathy following trauma (published in 2019) [10], and several local trauma and bleeding management guidelines, including those from the Czech and Slovak Republic [31], Sweden [32], Germany [26], Spain [27, 28] and the United Kingdom [30, 33] (Table 1).

**Table 1:** Summary of European guidelines for the treatment of trauma-induced coagulopathy

Guidelines	First-line treatment	Other recommendations	FCH indicated for acquired hypofibrinogenemia
**Europe**: *The European guideline on management of* *major bleeding and coagulopathy* *following trauma: fifth edition* [10]	Initial management of expected massive haemorrhage: **FFP** (or pathogen-inactivated FFP) (1C) or **FCH** (1C), with **RBC** **FCH** or **cryoprecipitate** for major bleeding with viscoelastic signs of fibrinogen deficit or a plasma fibrinogen level ≤ 1.5 g/L (1C) Initial **FCH** dose of 3–4 g is suggested; repeat doses guided by viscoelastic monitoring and fibrinogen laboratory tests (2C) Avoid **FFP** for hypofibrinogenaemia (1C)	**PCC** is recommended if fibrinogen levels are normal and viscoelastic monitoring indicates delayed coagulation initiation (2C) **rFVIIa** should be considered if major bleeding and coagulopathy continue despite all other attempts to control bleeding and best-practice use of conventional haemostatic measures (2C)	Yes
**Czech and Slovak Republic:** *Diagnosis and treatment of life-threatening bleeding in adult patients in intense and perioperative care (Czech-Slovak interdisciplinary recommended procedure)* [31]	Initial treatment with **FFP** and **ETP** (1:2, 1B) or **FCH** and **ETP** (1C) at appropriate values/levels Initial dose of at least 50 mg/kg **FCH** (1C) **FCH** when fibrinogen level < 1.5–2 g/L or by equivalent by viscoelastic testing (1C)	To maintain fibrinogen level at 2 g/L **PCC** (25–50 U/kg) is recommended when coagulation factor deficit is assumed (2C); risk/benefit should be assessed **rFVIIa** (90–100 µg/kg) to be considered if all standard measures fail and there is still life-threatening bleeding	Yes
**Sweden:** *Hemostasis and* *severe bleeding:* *Care program prepared by* *The Swedish Society for* *Thrombosis and Hemostasis working group* [32]	Early transfusion with **plasma** and **erythrocytes** (1:1) with platelet unit for every four units plasma/erythrocytes, if bleeding > 1–1.5 blood volume **FCH** (2–4 g) for > 1 blood volume	Subsequent fibrinogen supplementation based on viscoelastic testing; aim for fibrinogen level > 2 g/L and INR < 1.5	Yes
**Germany**: *Level 3 guideline on the treatment of patients with severe/multiple* *injuries (Polytrauma Guideline Update Group)* [26]	**FFP** is recommended for massive transfusion (4:4:1 FFP:pRBC:PLT ratio) **FCH** is recommended should a patient present with a fibrinogen level < 1.5 g/L (target fibrinogen level ≥ 1.5 g/L) **PCC** is recommended as a treatment option outside of VKA reversal if needed **FXIII** is also recommended if needed	N/A	Yes
**Spain:** *Spanish Consensus Statement on alternatives to allogeneic blood transfusion: the 2013 update of the "Seville Document*" [27]	Early **PCC** administration is recommended in non-VKA-treated patients presenting with coagulopathy **FCH** should be given if plasma fibrinogen < 2 g/L **rFVIIa** is recommended for severe refractory haemorrhge	N/A	Yes
**Spain**: *Multidisciplinary consensus document on the management of massive haemorrhage (HEMOMAS document)* [28]	**FFP** should be administered early for massive haemorrhage **FCH** should be given if plasma fibrinogen < 2 g/L **rFVIIa** is not recommended as a first-level option for massive haemorrhage	**PCC** is only recommended in non-VKA-treated patients if there is a risk of TACO or TRALI, or depending on the urgency of treatment and availability of FFP	Yes
**UK**: *A practical guideline for the haematological management of major haemorrhage (British Society of Haematology)* [30]	Present or expected massive haemorrhage: 1:1 ratio of **FFP**:**RBC** (1B*) Further **FFP** should be guided by laboratory tests with a transfusion trigger of PT and/or aPTT > 1.5 times normal (2C) If laboratory results are unavailable and bleeding continues: **FFP** and **RBC** in at least 1:2 ratio, before switching to blood product use guided by laboratory results (2C) Use of **FFP** should not delay fibrinogen supplementation if it is required (2C) Fibrinogen levels < 1.5 g/L: **cryoprecipitate** (2 pools) (1C)	**PCC** and **rFVIIa** are not recommended for major haemorrhage unless as part of a clinical trial (1D)	No
**UK**: *Blood transfusion and the anaesthetist: management of* *massive haemorrhage (Association of Anaesthetists of Great Britain and Ireland* *Membership)* [29]	Early infusion of **FFP** (15 mL/kg) Established coagulopathy (widespread microvascular oozing or inadequate haemostasis) indicated by fibrinogen < 1 g/L or PT/aPTT > 1.5 × normal): **FFP** at doses likely to correct coagulation factor deficiencies (≥ 30 mL/kg) Hypofibrinogenaemia unresponsive to FFP: cryoprecipitate is often recommended, but **FCH** (30–60 mg/kg) can achieve fibrinogen replacement more rapidly and predictably **PCC** and intravenous vitamin K (5–10 mg) for warfarin reversal	Some centres use **PCC** in certain clinical situations, such as liver disease and post-CPB; local protocols must be agreed in advance **rFVIIa** has been used for massive haemorrhage unresponsive to conventional therapy, but there may be a risk of arterial thrombotic complications; local protocols must be agreed in advance	No

aPTT, activated partial thromboplastin time; CBP, cardiopulmonary bypass; ETP, erythrocyte transfusion unit preparations; FCH, fibrinogen concentrate; FFP, fresh frozen plasma; Hb, haemoglobin; IV, intravenous; INR, international normalized ratio; MCF, maximum clot firmness; PCC, prothrombin complex concentrate; PLT, platelet; PT, prothrombin time; pRBC, packed red blood cells; RBC, red blood cells; rFVIIa, activated recombinant factor VII; TACO, transfusion-associated circulatory overload; TRALI, transfusion-related acute lung injury; VKA, vitamin K antagonist

For initial treatment of bleeding, the guidelines generally agree on the administration of TXA as soon as possible [10, 26–32], followed by an initial ratio-driven approach of FFP:red blood cells (RBC) or FCH:RBC to prevent/treat massive haemorrhage [10, 26, 30–32]. All European guidelines recommend fibrinogen supplementation with either FCH or cryoprecipitate when fibrinogen levels are low [10, 26–32], though the threshold fibrinogen level varies. The use of PCC for treatment of TIC in non-VKA-treated patients also varies between guidelines [10, 26–32], while activated recombinant factor VII (rFVIIa) is not recommended as a first-line therapy (Table 1).

## Current appraisal of the trauma guidelines across Europe

### Definition of coagulopathy

As discussed, a clear definition for TIC is lacking [8, 9]; current research is focused on determining laboratory-based haemostatic abnormalities, but the relationship between laboratory measurements (e.g. prothrombin time) and clinically evident coagulopathy (e.g. diffused oozing from injured and uninjured sites) is complex. In a prospective observational study, clinically evident coagulopathy was associated with poor outcomes in patients with trauma, but was rare compared with laboratory-defined coagulopathy [34]. In the absence of a clear TIC definition, it is difficult to ascertain patients who are clinically coagulopathic, and it is important to treat patients who are bleeding, not only those with laboratory-defined coagulopathy. Stratification of patients needing treatment is key, but an effective approach is yet to be defined.

Most published studies have used conventional coagulation tests, such as prothrombin time (PT) or international normalised ratio (INR), partial thromboplastin time (PTT) and fibrinogen to define TIC, but the precise thresholds and combinations of tests vary [35]. Peltan et al. suggested a definition of INR > 1.5, which provides a simple test to identify patients at increased risk of adverse outcomes [35], while Frith et al. suggested PT ratio (PTr) > 1.2 as a clinically relevant definition of TIC [9]. In contrast, Davenport et al. found that the viscoelastometric parameter CA5 ≤ 35 mm was able to identify more patients with TIC than PTr > 1.2 and could predict the need for massive transfusion [36]. This value was supported by another study, which suggested extrinsic pathway thromboelastometry (EXTEM) CA5 ≤ 40 mm and fibrinogen thromboelastometry (FIBTEM) ≤ 9 mm as markers for TIC [37].

These studies all use different thresholds, and a consensus is needed. We support a definition of TIC based on viscoelastometric measurements, which we define as EXTEM CA5 ≤ 40 mm and/or FIBTEM CA5 ≤ 9 mm. However, a definition of TIC is required that also encompasses the multifactorial nature of TIC, primarily endogenous anticoagulation, fibrinogen abnormalities, platelet dysfunction and endotheliopathy, but also the risk of additional factors, e.g. shock, hypothermia, metabolic acidosis, anaemia and haemodilution and exogenous anticoagulation [38].

We suggest a simple definition of TIC, that can be quickly and easily interpreted in clinical practice, especially in an emergency situation. Therefore, we recommend a grading system comprising three severity levels. These levels correspond to a patient with bleeding, shock and one of the following: TIC 1: fibrinogen level < 1.5 g/L; TIC 2: fibrinogen level < 1.5 g/L and INR > 1.5; TIC 3: fibrinogen level < 1.5 g/L and INR > 1.5 with platelet count < 100,000 × 10^9^/L (Table 2).

**Table 2:** Definition of TIC in a patient with bleeding and shock, using a grading system comprising three severity levels, based on fibrinogen level, INR and platelet count

Severity level	Definition
TIC 1 TIC 2 TIC 3	Fibrinogen level < 1.5 g/L Fibrinogen level < 1.5 g/L *and* INR > 1.5 Fibrinogen level < 1.5 g/L *and* INR > 1.5 with platelet count < 100,000 × 10^9^/L

INR, international normalized ratio; TIC, trauma-induced coagulopathy

While the TIC definition based on viscoelastometry may have the advantage of providing faster diagnosis [39], viscoelastic testing is not available in all hospitals. Furthermore, the viscoelastic approach addresses whether TIC is present or not; our grading system based on conventional parameters also informs on TIC severity. Therefore, the latter has the potential to guide haemostatic treatments, tailoring them to the patient’s severity. For the rapid measurement of INR in patients with suspected TIC, we suggest the use of a portable coagulometer instead of conventional laboratory tests [40].

### Criteria for coagulation and resuscitation therapy

Identifying patients who require an MTP is challenging based on the current guidelines, and current trauma scoring systems are complex in practice. There is a need to establish a simple trigger for the initiation of an MTP (e.g. clinically suspected or proven bleeding, hypofibrinogenaemia identified by viscoelastic testing, or clinical signs of shock). Indeed, hypofibrinogenaemia is predictive of the need for massive transfusion in trauma patients [41]. Viscoelastic tests are playing an increasingly important role in decision-making on when to initiate an MTP. In the absence of blood test results, the identification of early clinical signs (e.g. shock, low blood pressure, or base excess − 6 mmol/L, with the presence of a potential or verified bleeding source) could be used to guide supplementation; however, clinical parameters should not be taken in isolation, as early trauma care is highly dynamic.

We propose a simple set of criteria to guide when to administer an MTP in the majority of clinical trauma settings, not just specialised trauma centres, informed by clinical judgement and current protocols [42]. All the following criteria should be met:

1. Severe bleeding and clinical and/or laboratory signs of hypoperfusion/haemorrhagic shock;
2. Base excess − 6 mmol/L;
3. Haemoglobin ≤ 9 g/dL;
4. Blood pressure abnormalities (e.g. mean arterial pressure < 65 mmHg or systolic blood pressure < 100 mmHg), and
5. FIBTEM A5 < 10 mm.

However, we acknowledge that the target should be to avoid an MTP in patients.

Immediate administration of TXA.

Most guidelines recommend the administration of TXA as soon as possible to patients who are bleeding or at risk of major haemorrhage, generally within 3 h of injury [10, 26–32]. Indeed, the use of TXA has been supported by several studies [10], including the CRASH-2 [43] and CRASH-3 RCTs [44]. The CRASH-2 RCT reported a reduction in all-cause mortality and risk of death due to bleeding, in trauma patients who were treated with TXA within 8 h of injury versus those treated with placebo [43]. The CRASH-3 RCT subsequently reported a reduced risk of head injury-related death in patients with mild-to-moderate traumatic brain injury, who were treated within 3 h of injury with TXA versus placebo [44]. Furthermore, a meta-analysis of CRASH-2 and the postpartum haemorrhage WOMAN trial found immediate TXA treatment improved survival by more than 70%; thereafter, the survival benefit decreased by 10% for every 15 min of treatment delay, with no benefit after 3 h [45]. Therefore, we recommend the initial treatment of TIC with TXA.

### Role of early fibrinogen supplementation

Fibrinogen depletion occurs in TIC and progresses during trauma haemorrhage, with fibrinogen the first coagulation factor to reach critically low levels [46]. Trauma-related hypofibrinogenaemia is associated with poor outcomes and is an independent predictor of mortality [46–49]. It has been shown that for every 1 g/L increase in plasma fibrinogen at hospital admission, the odds of death decrease by 0.22 [46].

### FCH for the management of initial bleeding and coagulopathy

The European trauma guidelines recommend FFP:RBC or FCH:RBC (both grade 1C) in a ratio-driven approach, for the initial management of expected massive haemorrhage [10]. We suggest that FCH, and not FFP, be administered as part of the initial management of TIC, i.e. upon hospital admission and before coagulation tests have been performed; as fibrinogen declines early after trauma, FCH should be administered as soon as possible after hospital admission [41, 50]. Our suggestion to use FCH is based on its benefits over FFP. Indeed, in our experience, fast and targeted therapy is only possible with CFCs as the concentrations of coagulation factors, including fibrinogen in FFP are too low to increase, or possibly even maintain, already low plasma concentrations in a bleeding patient [15]. However, there is the caveat that further studies comparing FCH and FFP are needed.

### FCH for the treatment of hypofibrinogenaemia

For the first time, fibrinogen supplementation (FCH or cryoprecipitate) is recommended (grade 1C) in the European trauma guidelines for major bleeding with hypofibrinogenaemia (i.e. fibrinogen level ≤ 1.5 g/L). The use of FFP for hypofibrinogenaemia, or for patients without major bleeding, is not recommended (grade 1B and grade 1C, respectively) [10]. The German guidelines similarly recommend FCH administration when fibrinogen levels are < 1.5 g/L [26], while the British Society of Haematology guidelines recommend either the administration of cryoprecipitate for a fibrinogen level < 1.5 g/L [30], or the administration of cryoprecipitate/FCH, when FFP does not increase fibrinogen levels [29]. Furthermore, the recommended fibrinogen dose varies; e.g. 3–4 g FCH in the European trauma guidelines [10], 30–60 mg/kg FCH in the British guidelines [29]. An agreement on both the threshold fibrinogen level and the dose of FCH is required. We recommend a threshold fibrinogen level of < 1.5 g/L if there is ongoing bleeding/high bleeding risk.

Many European guidelines suggest goal-directed viscoelastic monitoring of coagulopathy and fibrinogen supplementation when there are viscoelastic signs of functional deficit [10, 26, 28, 30, 32]; however, only the Spanish guidelines (HEMOMAS) currently provide threshold levels (FIBTEM-maximum clot factor [MCF] < 7 mm) [28]. The British Society for Haematology guidelines on viscoelastic testing in major bleeding provide guidance on the use of these methods and interpretation of the results, but do not recommend specific FIBTEM trigger values for fibrinogen replacement in trauma, given the lack of high-quality data [51]. Several algorithms using ROTEM have been proposed; however, these suggestions are mostly based on retrospective data or expert consensus [52]. For example, a 2014 consensus conference on viscoelastic testing during resuscitation for trauma patients recommended fibrinogen supplementation with FIBTEM A10 < 10 mm, corresponding with FIBTEM MCF < 12 mm (plus abnormally low EXTEM A10 < 40 mm, corresponding to EXTEM MCF < 50 mm) [53]. Several institutions have also published their viscoelastic-based algorithms for TIC management [17, 54].

We suggest that FCH should be administered as first-line therapy for the treatment of hypofibrinogenaemia in TIC. Overall, a patient-individualised fibrinogen dosing regimen, using viscoelastic testing or a weight-based calculation, may be more appropriate than stipulating a specific dose; however, it is acknowledged that weighing patients in an acute setting is challenging.

In an emergency situation, a standard dose of FCH (3–4 g) [10] may be administered in the presence of hypofibrinogenaemia, to prevent delay and stabilise the clotting process [55]. Subsequently, the dose may be adjusted per the results of viscoelastic testing (if available), e.g. 6 g for FIBTEM A5 0 mm; 5 g for FIBTEM A5 1–4 mm; 4 g for FIBTEM A5 5–6 mm; 3 g for FIBTEM A5 7–8 mm; or 2 g for FIBTEM A5 9–10 mm [56], or a weight-based dose equivalent if appropriate. The main focus should be on dynamic blood loss and ongoing bleeding, while viscoelastic testing to diagnose fibrinogen deficiencies should encompass viscoelastic tests other than FIBTEM, often with different threshold levels [57]. In addition, if a haemorrhagic patient presents with fibrinogen levels just above the threshold for initiating fibrinogen supplementation, we suggest that fibrinogen should still be administered. After initial fibrinogen supplementation, and if bleeding continues, further viscoelastic (FIBTEM) testing should be conducted after ~ 30 min, to verify the impact of the fibrinogen supplementation and to identify other causes for bleeding that may have previously been masked by hypofibrinogenaemia.

### Use of PCC for the management of TIC

Reduced thrombin generation has been associated with increased mortality in trauma patients, suggesting that PCC treatment may be beneficial [58]. An observational study of major trauma showed that PCC administration was associated with increased endogenous thrombin potential and lower antithrombin levels versus control groups, though not indicated with laboratory tests [59], while another observational study with trauma patients found that PCC and FFP administration was associated with lower mortality, compared with FFP alone [60]. However, data supporting the use of PCC in TIC management are currently limited [61].

The recommendations for PCC administration for TIC vary by country (Table 1). The European trauma guidelines suggest that PCC is given to bleeding patients with delayed coagulation initiation (using viscoelastic testing), if fibrinogen levels are normal [10], while the Spanish guidelines recommend or suggest PCC as a treatment outside of VKA reversal, if required or under specific circumstances [27, 28]. According to the guidelines from the Association of Anaesthetists of Great Britain and Ireland, some centres may use PCC in specific clinical situations (e.g. liver disease) and local protocols must be agreed in advance [29]. However, in the British Society of Haematology guidelines, PCC is not recommended for major haemorrhage unless as part of a clinical trial [30], and in one expert’s clinical experience, PCC is not commonly administered for trauma-related bleeding in Sweden [32].

Overall, impaired thrombin generation is not considered a problem in the early stages of trauma-related bleeding management, as thrombin levels are often increased following trauma. Indeed, studies have found greater thrombin generation in trauma patients compared with healthy controls [58, 62]. In addition, there is no reliable laboratory test to verify absolute factor deficiencies in prothrombin complex coagulation factors; thus, other contributing factors, such as hypofibrinogenaemia and hyperfibrinolysis, should be managed first and the severity/risk of ongoing bleeding determined, before PCC administration.

### The TIC treatment sequence

**Fig. 1:**
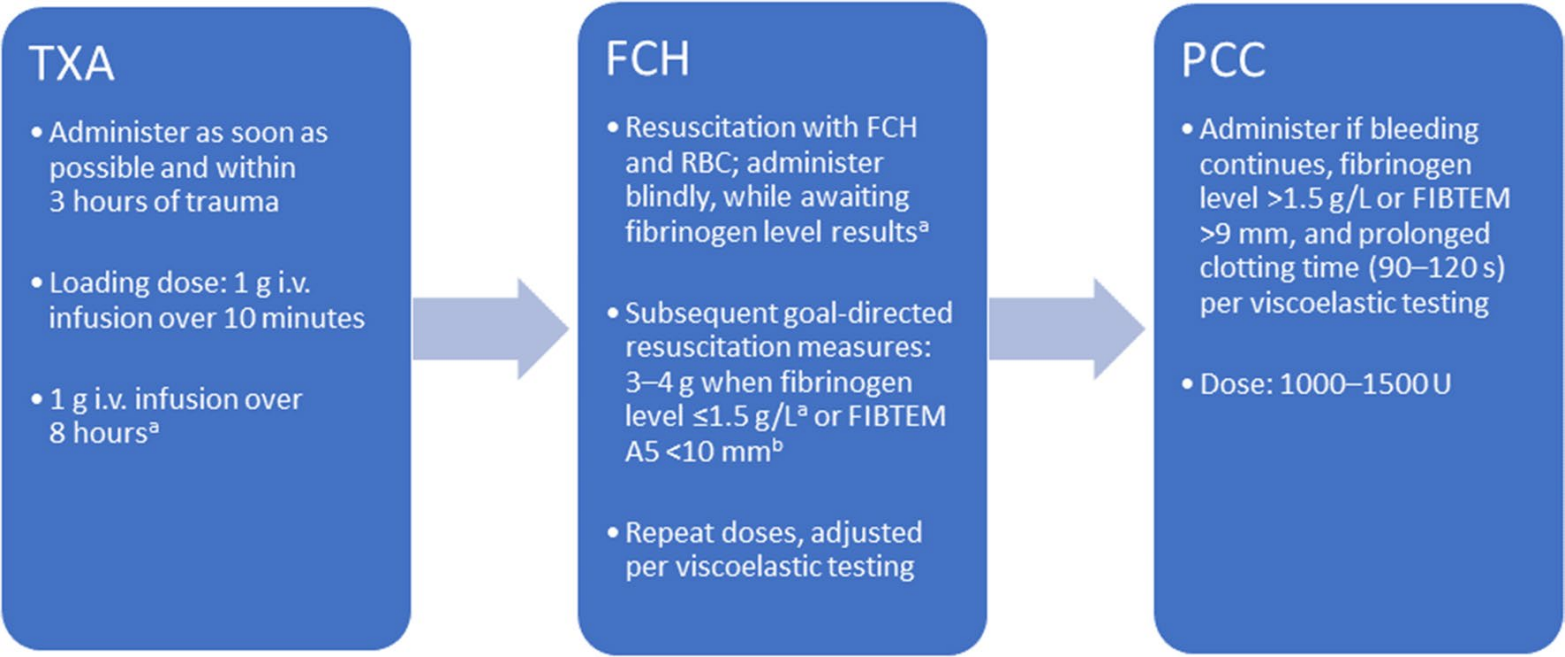
Recommended treatment sequence massive bleeding and trauma-induced coagulopathy. ^a^Informed by the fifth edition of the European trauma guidelines [10]. ^b^Viscoelastic tests other than FIBTEM can be used. FIBTEM A5, clot amplitude 5 min after clot formation; FCH, fibrinogen concentrate; i.v., intravenous; PCC, prothrombin complex concentrate; TXA, tranexamic acid

We believe a step-wise approach to the treatment for trauma-related bleeding allows for individualised therapy, and avoids overtreatment and unnecessary allogeneic transfusion. Therefore, we suggest the initial administration of TXA, followed by FCH, and lastly PCC if bleeding continues (Fig. 1), with weight-adjusted doses if possible. However, we acknowledge that the recommendation for the use of PCC in a patient with a normal fibrinogen level (> 1.5 g/L), but with continued bleeding and a prolonged clotting time is a weak recommendation, and is listed as a second-line treatment recommendation in the fifth European trauma guidelines (Grade 2C) [10]. PCC recommendations also vary by country, as described below and in Table 1; we recommend that PCC should be administered only in the presence of a prolonged clotting time.

### Overcoming current barriers to effective CFC administration and future initiatives

#### Improved medical education

A survey of real-world trauma practice revealed heterogeneity in both the treatment of trauma patients and in local resources across Europe, as well as frequent deviations from the European guidelines [63]. This highlights a need for improved awareness of guideline recommendations when treating bleeding in trauma. Compliance may be improved with standardised questionnaires or simple guides, such as a handbook of algorithm templates on trauma and other bleeding scenarios. A number of educational solutions are also proposed, including the development of accredited online courses for the management of critical haemorrhage in different clinical scenarios (similar to the Advanced Trauma Life Support teaching programme [64] and the HEMACRIT course for the management of massive and/or critical haemorrhage [65]), or materials to educate clinicians at congresses.

Another barrier to implementing the current guidelines is when to use CFCs over FFP, as the same level of recommendation (grade 1C) is given in the fifth European trauma guidelines for ratio-driven (i.e. fixed ratio of FFP and RBCs) and coagulation factor-driven (i.e. FCH and RBCs) strategies for initial coagulation resuscitation [10]. The absence of CFCs from local treatment algorithms can also limit CFC use; a solution may be the development of local guidelines that account for country-specific indications and local availability of CFCs. A key component is the need for prior approval of CFC use by hospital pharmaceutical committees; in some countries such committees are key decision makers for the inclusion of CFCs in an MTP.

#### The requirement for additional data on CFCs

There is need for more adequately powered and well-designed RCTs that directly compare FFP and CFC therapies. However, these studies are challenging due to the ethical issues in withholding CFC treatment in patient subgroups. Local/national audits that survey haemostatic management may clarify issues with CFC use and facilitate improvements in trauma management.

#### Perceived additional costs with CFCs

FCH was recently shown to be non-inferior to cryoprecipitate, in terms of transfusion requirements, for the treatment of bleeding in patients after cardiac surgery [66]. Other ongoing studies may shed additional light on the effectiveness of FCH versus cryoprecipitate in trauma and other clinical settings [56, 67]; however, the overall presumption by hospital boards and pharmacies that FFP and cryoprecipitate are cheaper treatment options than CFCs, is a barrier to effective CFC therapy.

Cost-effectiveness analyses are required to directly compare the cost of FCH with FFP and/or cryoprecipitate, considering local regulations, specialties and conditions. A recent US model predicted that FCH would be more expensive than cryoprecipitate in adult trauma, even after adjusting for wastage and technologist time [68]. However, many other costs are involved when using blood products (e.g. blood banks), which are often underestimated and vary by institution and country [69]. Clarification of the actual costs and reimbursement policies in different countries would be beneficial to identify barriers in CFC use, and a local or national consensus on the financial support for haemostasis management is needed. In addition, other factors such as the preservation of blood reserves and the costs in treating blood-borne infections potentially transmitted via blood products should be considered when assessing the relative cost-effectiveness, with the risk lower for CFCs than cryoprecipitate or FFP [70]. A prospective analysis that evaluated the transition from a blood product- to an FCH-based trauma protocol across two trauma centres recorded a cost saving of 23% over approximately 2 years [42].

#### Perceived risk of thromboembolic events

The perceived risk of thromboembolic events when using FCH is another barrier to its use. However, while FCH increases the level of plasma fibrinogen, it does not increase above the threshold fibrinogen level in a normal, acute-phase response, suggesting it is unlikely to increase prothrombotic status [71]. In a meta-analysis of 14 RCTs with adult and paediatric surgical patients, there was no difference in the number of thromboembolic complications between patients who received FCH compared with placebo/comparator [72]. Similarly, reviews of clinical trial [73] and pharmacovigilance [74] data have found the risk of thrombosis is low with FCH.

#### Access to goal-directed coagulation methods

Lack of access to viscoelastic testing in some countries is a barrier to an individualised treatment approach. In low-to-moderate-income countries, organisations responsible for national blood management could provide POC devices for trauma centres to overcome this barrier. A recent pilot study that investigated the implementation of a new viscoelastic-based treatment algorithm across four European trauma centres found that ROTEM results were available significantly earlier, and identified more patients with coagulation abnormalities than conventional laboratory tests [75]. These results suggest that the introduction of new treatment algorithms is feasible and may lead to more rapid and precise coagulation management.

#### CFC reconstitution and preparation times

Another potential barrier to CFC use is reconstitution and preparation times, potentially leading to a delay in treatment administration. Improvements in FCH administration may facilitate increased use, such as the development of a ready-to-use syringe. Reconstitution of CFCs could be accelerated by using greater quantities of CFC in each vial; for example, some FCH formulations are available with a greater fibrinogen content per vial [25, 43, 76]. In the authors’ institutions, delays to the administration of blood components and CFCs have also been reduced with the provision of refrigerated packs of blood products (RBCs, FFP and platelets) and CFC kits (for example, containing 4 g TXA, 3 g FCH and 1500–1800 U PCC) at key hospital locations.

## Conclusions

The management of TIC remains challenging, with a high degree of variability in recommendations for the treatment of patients with major trauma bleeding in local and supranational guidelines. Indeed, the development and implementation of guidance can be challenging in some countries, and the lack of a clear definition of TIC may hinder the administration of effective treatment. Our suggestion for a simple definition of TIC may be helpful to both trigger and guide the initiation of haemostatic therapy.

CFCs, particularly FCH, play a major role in the early management of trauma, but the evidence base needs to be strengthened. A number of initiatives may improve TIC management. Better medical education is of major importance, as well as the generation of new and stronger data and improved access to viscoelastic testing. The key take-home messages are that TIC should be considered early in all major trauma bleeding patients, and when following protocols, the best practice is to be proactive and preventative; however, this can be difficult to justify from a cost perspective. Addressing these issues may help to contribute to the ultimate goal of improving patient care.
